# Cost-effectiveness of Practice Team-Supported Exposure Training for Panic Disorder and Agoraphobia in Primary Care: a Cluster-Randomized Trial

**DOI:** 10.1007/s11606-020-05658-9

**Published:** 2020-01-21

**Authors:** Christian Brettschneider, Jochen Gensichen, Thomas S Hiller, Jörg Breitbart, Ulrike Schumacher, Karoline Lukaschek, Tobias Teismann, Jürgen Margraf, Hans-Helmut König

**Affiliations:** 1 Department of Health Economics and Health Services Research, Hamburg Center for Health Economics, University Medical Center Hamburg-Eppendorf, Hamburg, Germany; 2 Institute of General Practice and Family Medicine, University Hospital of Ludwig-Maximilians-University Munich, Munich, Germany; 3 Institute of General Practice and Family Medicine, Jena University Hospital, Jena, Germany; 4 Centre for Clinical Studies, Jena University Hospital, Jena, Germany; 5 Mental Health Research and Treatment Center, Ruhr-Universität Bochum, Bochum, Germany

**Keywords:** self-management, cost-effectiveness, mental health, primary care

## Abstract

**Background:**

Primary care is the main treatment setting for panic disorder and should be supplemented by collaborative care programs. However, shortage of mental health professionals prevents collaborative care programs from being effectively implemented. The PARADISE study showed the efficacy of a self-managed, cognitive-behavioural therapy (CBT)-oriented exposure training for patients with panic disorder with or without agoraphobia in primary care delivered by the family practice team.

**Objective:**

To assess the cost-effectiveness of the PARADISE intervention.

**Design:**

Cost-effectiveness analysis from the societal perspective based on data from a cluster-randomized controlled trial over a time horizon of 12 months.

**Participants:**

Four hundred nineteen adult panic disorder patients with or without agoraphobia.

**Interventions:**

A self-managed, CBT-oriented exposure training for patients with panic disorder with or without agoraphobia in primary care delivered by the primary care practice team in comparison to routine care.

**Main Measures:**

Total costs from the societal perspective. Direct costs and disease-specific costs. Quality-adjusted life years based on the EQ-5D-3L. Incremental cost-effectiveness ratios and cost-effectiveness acceptability curves.

**Key Results:**

Patients in the intervention group caused lower costs (mean, €1017; 95% confidence interval [-€3306; €1272]; *p* = 0.38) and gained on average more QALY (mean, 0.034 QALY (95% confidence interval [0.005; 0.062]; *p* = 0.02). Therefore, the intervention dominated the control treatment. The probability of cost-effectiveness of the intervention at a willingness-to-pay margin of €50,000 per QALY was 96%. Results from supplementary analyses considering direct or disease-specific costs instead of total costs showed comparable results.

**Conclusion:**

The PARADISE intervention is cost effective. This conclusion is valid for total costs, generic health care (direct) costs, disease-specific health care costs.

**Trial Registration:**

German Clinical Trials Register: DRKS00004386

Current Controlled Trials: ISRCTN64669297

**Electronic supplementary material:**

The online version of this article (10.1007/s11606-020-05658-9) contains supplementary material, which is available to authorized users.

## INTRODUCTION

Panic disorder is defined by recurrent unexpected panic attacks and worries about future attacks and/or the avoidance of specific situations.^1^ Panic attacks are characterized by an abrupt surge of fear in combination with symptoms like sweating, pounding heart or shortness of breath.^1^ Patients with agoraphobia demonstrate marked fear of specific situations like using public transport, visiting marketplaces, theatres or shops or being in a crowd. As a consequence, patients avoid these situations.^1^ Panic disorder and agoraphobia often co-occur (30–60%).^2^

The 12-month prevalence of panic disorder in Germany is 2% (95% confidence interval 1.6–2.5%).^3^ Women are more frequently affected than men (women 2.8% [2.2–3.6%]; men 1.2% [0.8–1.8%]).^3^

Studies have shown that panic disorder is associated with a loss of physical and mental quality of life^4, 5^ and a high economic burden.^6^ Furthermore, evidence suggests that a misinterpretation of bodily sensations and health anxiety could increase health care utilization.^7, 8^

Primary care is the main treatment setting for patients with anxiety disorders.^2, 9, 10^ Collaborative care programmes are widely employed in their treatment.^11^ However, due to a shortage of mental health professionals and consequently long waiting times for an appointment,^12^ collaborative care programmes incorporating mental health professionals are difficult to implement.

The aim of the “Patient Activation foR Anxiety DISordErs” (PARADISE) study was to implement a self-managed, cognitive behavioural therapy (CBT)-oriented exposure training for patients with panic disorder with or without agoraphobia in primary care delivered by the practice team. The effectiveness of the PARADISE intervention has already been demonstrated.^13^

In this article, we present evidence on the cost-effectiveness of the PARADISE intervention in comparison to usual primary care.

## METHODS

### Sample

The PARADISE study was a cluster-randomized controlled trial comparing a practice team-supported exposure training for panic disorder and agoraphobia in primary care to usual care. Primary care practices registered with the regional Association of Statutory Health Insurance Physicians (Thuringia, Germany) were invited to participate. Inclusion criteria for patients were age 18 years or older; a diagnosis of panic disorder with (ICD-10: F40.01) or without agoraphobia (F41.0); a score of 8 or higher on the Overall Anxiety Severity and Impairment Scale (OASIS)^14^ and a score of 2 or higher on the Patient Health Questionnaire (PHQ) panic module.^15^,^16^ Exclusion criteria were acute suicidal ideation, psychotic or substance-related disorders, severe somatic diseases, pregnancy or current anxiety-specific psychotherapy.

Primary care practices were randomized by location (urban/rural) in a 1:1 ratio. For further information, please see Gensichen J et al.^13^

### Intervention

All practice teams were instructed in diagnostics and treatment standards of panic disorder in a 2-h session. Practice teams consisted of primary care physicians (PCP) and examined medical assistants (MA), employees with a specific vocational qualification.^17^ PCP in both groups were free to initiate any medical treatment or refer the patient to other health care professionals (routine care).

In the intervention group (IG), PCP executed the PARADISE programme consisting of four structured appointments over a period of 23 weeks. The first three appointments comprised three major interventions of cognitive-behavioural therapy (psychoeducation; interoceptive exposure exercises, and situational exposure exercises). The fourth appointment provided time to reflect the patient’s experiences. In addition, patients were requested to perform practice exposure exercises at least two times a week at home. As support, patients received a workbook. Medical assistants (MA) performed the clinical monitoring of the process by ten telephone contacts. In these contacts, the MA assessed current anxiety severity and adherence to exercises.

## ASSESSMENT OF COSTS AND EFFECTS

### Data Collection

Data were collected at baseline (T0), after 6 months (T1), and after 12 months (T2) by a self-reported questionnaire. In addition to resource utilization and preference-based health-related quality of life (EQ-5D-3L^18^), we collected data on sociodemographics, severity of anxiety (Beck Anxiety Inventory [BAI]^19, 20^) and comorbidities.

### Costs

#### Questionnaire of Service Utilization and Costs

We chose a narrow definition of the societal perspective in this study, focussing on the most important and common cost categories.^21^ We considered direct costs, representing the resource utilization caused in the formal health care sector (inpatient services, outpatient services, medications and professional home care), in the informal health care sector (informal unpaid care) and by the intervention itself. Furthermore, we considered indirect costs in the market sector, i.e. productivity losses caused by sick leave and by contacts with health care professionals during working hours.^22^

#### Unit Costs

Unit costs constituting the foundation of cost calculation are presented in Table 1. Reference year for cost calculation was the 2012. Cost are presented in Euro (€).

**Table 1:** Unit Costs Considered in the Calculation of Costs

Sector	Service/goods	Units	Monetary values (unit costs)
Inpatient services	General hospitals, psychiatric hospitals and hospitals for rehabilitation	Days	Type specific mean rates^20^
Outpatient physician services	GP, psychiatrist, other specialist	Contacts	Type specific mean rates^20^
Outpatient psychotherapist services	Psychotherapist	Contacts	Reimbursement schedule^20^
Medication	Product	Quantity	Official pharmaceutical index (Rote Liste)^21^
Home care	Professional ambulatory care	Hours	Type specific wage^20^
	Informal care	Hours	Type specific wage (replacement cost approach)^20^
Indirect costs	Productivity losses	Hours	Gross income plus non-wage labour costs^22^

All direct cost categories were valued based on the German opportunity cost based, standardized unit cost catalogue by Bock et al.,^23^ except for medication, which was valued based on the Rote Liste, an official German pharmaceutical index.^24^ Indirect costs were calculated based on the human capital approach by using gross income plus non-wage labour costs.^25^

Cost were not discounted as the time horizon of this study was one year.

#### Intervention Costs

The intervention consisted of structured sessions conducted by the PCP and of clinical monitoring by telephone conducted by the MA. As patients had the opportunity to make use of less or more services, we calculated the intervention costs based on the effective utilization of services.

One interventional session conducted by the physician was valued by €60.66. Derivation: We chose the opportunity cost approach. A session lasted 27 min on average. A regular contact with a primary care PCP in Germany has a median duration of 9 min.^26^ This means that in the same time a physician needed to perform the intervention, he could have treated three patients. The societal opportunity costs for an average contact with a PCP are €20.22.^23^

One clinical monitoring contact by the MA was valued with €8.13. Derivation: We chose the opportunity cost approach. The gross income plus non-wage compensations of an employee in the German health care system is €32.57 per hour. One average telephone contact for clinical monitoring lasted 15 min. Therefore, we calculated a fourth of the hourly gross income plus non-wage compensations.^25^

#### Effects

We performed our analyses based on quality-adjusted life years (QALY).

To calculate QALY, preference-based quality of life and the duration a patient lived in this health state has to be measured.

Preference-based health-related quality of life (HRQL) was measured by the three-level version of the EQ-5D (EQ-5D-3L).^18^ The EQ–5D-3L is a generic HRQL questionnaire. It is composed of five items assessing current problems in the dimensions mobility; self-care; usual activities; pain/discomfort; and anxiety/depression.^18^ Answers are coded as follows: 1—no problems; 2—moderate problems; 3—extreme problems. The EQ-5D-3L can describe 243^27^ health states. For all 243 health states, a utility score (EQ-5D index score) was calculated. In the present study, EQ-5D index scores from the UK were used.^28^ These EQ-5D index scores range from − 0.594 (worst health state) to 1 (best health state). The EQ-5D has been validated in populations with anxiety disorders.^29^

QALY were calculated by multiplying the EQ-5D index score with the days the patient lived in this health state. No patients died over the course of the study. We calculated QALY for each 6-month observation period (T0-T1; T1-T2) separately and computed the total 12-month QALY by adding up the periodical values.

Effects were not discounted as the time horizon of this study was 1 year.

### Statistical Analysis

Missing values were imputed on an item level by multiple imputation using chained equations (MICE).^30, 31^ The highest share of missing values on the item level was 30%. At baseline, 74% of participants had no missing values, at T1 63%, and at T2 57% (a comprehensive presentation of missing values per time point and trial arm can be found in Table 5 in the online Appendix). In total, we created 50 datasets based on 93 variables assessed at baseline, T1, and T2.

### Primary Analysis

The primary analysis was based on total costs from the societal perspective and QALY. Adjusted differences in mean costs and QALY after 12 months were analysed by means of linear mixed models with bootstrapped standard errors (1000 replications). Variable of interest in these models was the group variable (0 = control group; 1 = intervention group). To account for the clustered structure of the data we included a random effect for primary care practice. The adjustment for baseline differences is recommend in the literature.^32, 33^ For this reason, we adjusted the models for variables showing group differences with a *p* value ≤ 0.1 at baseline. This applied to the retirement status, costs (analyses of single cost categories were adjusted for the specific baseline value. The analyses of QALY were adjusted for the total costs at baseline) and health-related quality of life as well as for the presence of joint diseases, depression and somatoform disorders. Additionally, we adjusted the models for age and gender as commonly associated factors of resource utilization and for the presence of gastrointestinal diseases, which showed a rather low *p* value and might possess a specific influence on the utilization of health care resources of patients with panic disorder. Adjusted cost or effect differences in Table 4 represent the coefficients of the group variable.

In the assessment of cost-effectiveness, we calculated incremental cost-effectiveness ratios (ICER) based on 12-month costs and QALY.

As the ICER is a point estimate and does not consider the uncertainty within the data, we calculated a cost-effectiveness acceptability curve (CEAC) by conducting a series of net-benefit regressions (NBR) using different willingness-to-pay (WTP) margins.^34^ NBR were performed by means of linear mixed models with a random effect for primary care practice and bootstrapped standard errors (1000 replications).

We controlled for the same covariates mentioned in the cost analysis. WTP margins ranged from €0 per QALY to €150,000 per QALY.

### Supplementary Analyses

First, we included only health care costs (direct costs) into the cost-effectiveness analyses. Second, we considered mental health-specific costs (stays in psychiatric hospital, outpatient contacts with psychiatrists or psychotherapists, utilization of psychopharmacological agents) exclusively. All analyses were conducted with Stata 15.1. (StataCorp, College Station, TX).

## RESULTS

### Baseline Characteristics of the Study Population

The IG contained 230 patients, the CG 189 patients. Table 2 shows the baseline characteristics of IG and CG. Patients in the IG were significantly less frequently retired, had higher costs, lower HRQL values and suffered more frequently from depression and joint diseases.

**Table 2:** Baseline Characteristics and Group Comparison of the Imputed Sample (*n* = 50)

Characteristic	Intervention group (*n* = 230)	Control group (*n* = 189)	*p* value
Age (years)			
Mean (SE)	46.09 (0.93)	46.25 (1.07)	0.91
Female: %	72.17	76.72	0.29
Single: %	37.39	39.68	0.63
Cohibitants			
Mean (SE)	2.52 (0.08)	2.33 (0.08)	
Education (years)			
Mean (SE)	11.20 (0.22)	10.87 (0.18)	0.25
Employed: %	63.26	57.51	0.26
Retired: %	15.11	25.40	0.01
Severity of anxiety (BAI)			
Mean (SE)	28.22 (0.83)	28.20 (0.92)	0.98
Total costs (€)			
Mean (SE)	6021.34 (692.34)	4275.66 (471.68)	0.04
EQ-5D Index			
Mean (SE)	0.568 (0.019)	0.619 (0.020)	0.07
Comorbidities: %			
Pulmonary diseases	13.48	15.34	0.59
Joint diseases	15.22	8.99	0.05
Intestinal diseases	24.35	20.11	0.30
Cardiovascular diseases	37.39	39.15	0.71
Depression	26.09	17.99	0.05
Somatic symptom disorder	11.30	6.35	0.07

*BAI* Beck Anxiety Inventory

In total, patients were on average 46 years old; the majority was female and lived in a partnership. More than half of the patients were employed. The mean disease severity was 28 points on the BAI indicating a moderate anxiety disorder. However, 45% of the patients suffered from severe anxiety (data not shown). HRQL was markedly reduced. The most common comorbidities were cardiovascular diseases (38% of patients), intestinal diseases (22%), and depression (22%).

### Primary Analysis

#### Comparison of Costs and Effects

Unadjusted costs and QALY are presented in Table 3. Adjusted differences in costs and QALY after 12 months are presented in Table 4. Total costs in the IG were smaller than in the CG (-€1017 [SE: €1168]). Furthermore, there was a tendency to lower costs in the intervention group in most categories, except for intervention costs. No differences in costs except for the intervention costs (*p* < 0.01) were statistically significant.

**Table 3:** Unadjusted Costs and QALY After 12 months

Category	Intervention group (*n* = 230)		Control group (*n* = 189)	
	Mean	SE	Mean	SE
Direct costs	4369.43	514.16	4239.46	568.69
Inpatient services	1993.82	361.40	2208.98	449.34
Psychiatric hospital	653.85	264.20	837.16	324.70
Outpatient services	854.91	74.34	776.03	62.29
Psychiatrist	42.29	8.47	45.24	8.87
Psychotherapist	358.46	63.95	311.64	52.09
Medication	508.46	71.85	643.69	134.51
Psychopharmaceuticals	186.94	26.09	150.17	20.80
Professional care	20.79	11.87	2.63	3.44
Informal care	728.54	207.68	608.14	180.05
Intervention*	262.91	8.05	0	0
Indirect costs	3930.64	652.86	3637.65	567.67
Total costs	8300.07	906.27	7877.11	958.78
Total mental health costs	1504.45	279.07	1344.21	339.62
QALY	0.675	0.015	0.665	0.016

*QALY* quality-adjusted life year. *Significant difference between intervention group and control group (*p* < 0.05)

**Table 4:** Adjusted Differences in Costs and QALY After 12 months

Category			95% confidence interval		
	Mean	SE	Lower limit	Upper limit	*p* value
Direct costs	− 679.93	710.28	−2072.09	712.22	0.34
Inpatient services	− 618.32	581.94	− 1758.94	522.29	0.29
Psychiatric hospital	− 365.06	417.85	− 1184.03	453.92	0.38
Outpatient services	− 66.39	89.04	− 240.91	108.13	0.46
Psychiatrist	− 12.93	11.78	− 36.03	11.16	0.27
Psychotherapist	− 71.41	78.47	− 225.21	82.39	0.36
Medication	− 64.41	83.30	− 227.69	98.86	0.44
Psychopharmaceuticals	22.83	26.77	− 29.64	75.29	0.39
Informal care	− 5.28	248.46	− 492.25	481.70	0.98
Intervention*	254.64	10.81	233.45	275.83	< 0.01
Indirect costs	− 369.29	716.76	− 1774.14	1035.56	0.60
Total costs	− 1017.04	1168.01	− 3306.33	1272.26	0.38
Total mental health costs	− 156.93	436.08	− 1011.65	697.78	0.72
QALY*	0.034	0.015	0.005	0.062	0.02

*QALY* quality-adjusted life year. *Significant difference between intervention group and control group (*p* < 0.05)

Statistically significant results in favour of the IG were found for QALY. Patients in the IG gained on average 0.034 additional QALY (SE: 0.015; *p* = 0.02) compared to patients in the CG. As the intervention was cost-saving and more effective, the point estimate for the ICER indicated dominance of the intervention.

#### Probability of Cost-effectiveness

**Fig. 1:**
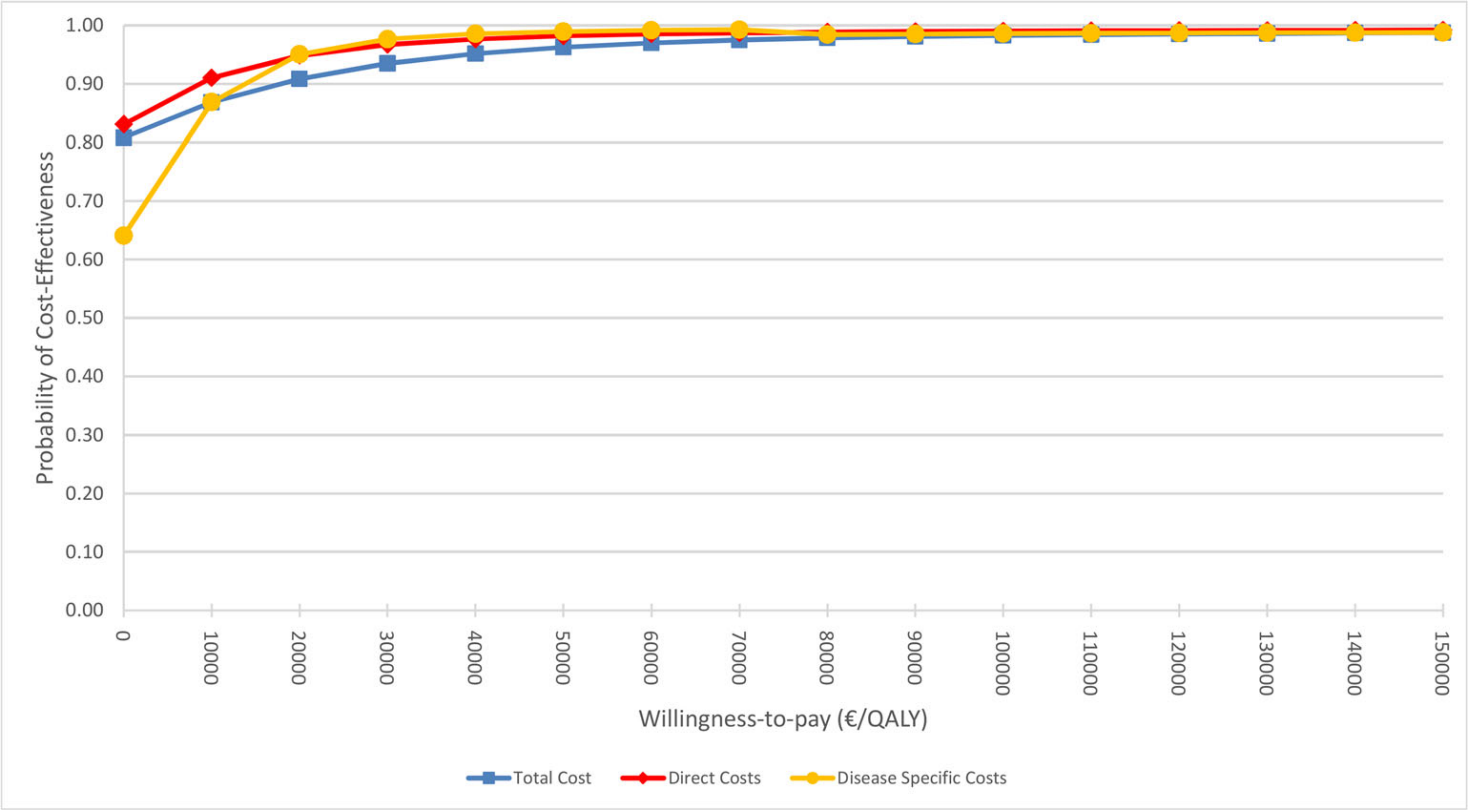
Cost-effectiveness acceptability curves based on total, direct, and disease-specific cost.

Figure 1 presents the CEAC based on the WTP for one QALY. If WTP was €0/QALY, the probability of cost-effectiveness is 81%. At a WTP of €50,000/QALY, the probability of cost-effectiveness was 96%.]-->

### Supplementary Analyses

#### Direct Costs

Direct costs accounted for 52% of total costs in both groups (Table 3). Patients in the IG caused lower adjusted direct costs than patients in the CG (€-679 [SE: €710]). Yet, this difference was not statistically significant.

The CEAC started at a lower level of probability than in the primary analysis (QALY 76% [Fig. 1]). However, at a WTP of €50,000 per QALY, the probability of cost-effectiveness was 95%.

#### Mental Health-Specific Costs

In both groups, the share of mental health-specific costs in total costs was approximately 18% (Table 3). After adjustment, the intervention costs were the only cost category with statistically significant differences. However, the IG showed a tendency to lower costs for nearly all services. However, costs for psychopharmaceuticals were slightly higher in the IG, yet not statistically significant.

The CEAC showed lower probabilities of cost-effectiveness at a WTP of €0/QALY (64%; Fig. 1) in comparison to the other CEAC. However, the probability increased reached 98% at a WTP of €50,000/QALY.

## DISCUSSION

The PARADISE intervention addressed the treatment of panic disorder and aimed at the reduction of symptom severity. The effectiveness of the intervention has already been shown.^13^ Therefore, the aim of our analysis was to provide evidence concerning the cost-effectiveness of the PARADISE intervention.

Our results confirm that the intervention is cost-effective. At a WTP of €50,000/QALY—a frequently adopted margin for cost-effectiveness^27, 35–38^—the probability of cost-effectiveness was at least 95% in the primary and supplementary analyses. More precisely, we found, on the one hand, no statistically robust evidence that the intervention was cost-saving in total costs or in single cost categories. The main finding that leads us to this conclusion is that there was no statistically significant (*p* ≤ 0.05) cost difference between the groups. Even though there was a probability of 81% of cost-effectiveness at a margin of 0€/QALY, this result should not be misinterpreted. The probability of 81% implies that there is a 19% margin of error in assuming that the intervention is cost-saving. This margin of error precludes the assumption that the intervention is cost-saving. However, we observed a significantly higher number of QALY gained by the intervention. Summarizing, under consideration of the significantly better health effects and the high probability of cost-effectiveness in the uncertainty analysis, it is justified to conclude that the intervention offers good value for money, even though it does not lead to significant cost savings.

Comparing our results with results from other studies is difficult. Few studies evaluated comparable interventions and in most of these studies, an economic evaluation was not conducted. An exemption is the CBT-based coordinated anxiety learning and management (CALM) treatment model for patients with different anxiety disorders.^39, 40^ Non-expert care managers in co-operation with PCP delivered the intervention. The economic evaluation of CALM^40^ identified a tendency to cost-savings in most categories. Yet, there were two categories with higher costs. First, costs for psychopharmacology were slightly higher in the CALM group. This is in line with our findings from PARADISE and might be due to the structured character of both programs where physicians might be more aware of the needs of patients. Second, costs for outpatient physician services were higher in the CALM group, whereas we identified slightly lower costs. Yet, this divergence is simple to explain as in the economic evaluation of CALM there was no specific category for intervention costs. As the intervention was delivered in primary care, interventions costs were part of the costs of outpatient physician services. In our study, intervention costs were a separate cost category.

Looking closer at outpatient costs, we observed significantly increased costs for primary care services in the IG (mean difference: €53.38 [SE: €19.10]; *p* < 0.01). This increase was independent from the intervention costs. We know from previous cost-of-illness studies that panic disorder not only increases mental health-specific costs but also general health care costs.^41^ However, costs for general and mental health-specific health care were decreased by the PARADISE intervention. This suggests that for patients with panic disorder an investment in primary care might lead to cost savings in other health care sectors. This relationship should draw greater attention in future research.

The foremost limitation of our randomized study is the different size of the IG (*n* = 230) and the CG (*n* = 189). An explanation for this is that this trial was not blinded. Practice teams recruited participants after the randomization. These circumstances increase the risk of a selection bias. Sociodemographic characteristics like age, gender or education were well balanced between groups. Baseline disease severity was comparable as well. However, there was an imbalance in total costs and health-related quality of life. This indicates that the patients’ need for treatment and psychological strain was higher in the IG than in the CG. We assume that practice teams in intervention practices had a high motivation to recruit participants, as they were able to offer patients a disease-specific, innovative treatment. To compensate these imbalances, we adjusted for the corresponding variables. Furthermore, we performed a difference-in-difference analysis of our results to examine if it led to other results (data not shown). The results were comparable. Considering this, we conclude that our results are robust. A further limitation is in the narrow interpretation of the social perspective. Due to the non-consideration of e.g. voluntary work or patient time for reasons of necessity to keep the responder burden low, our results might be biased. However, based on a review by Drost et al., our interpretation of the societal perspective is in line with the major part of the literature and hence our results can be assumed to be suitable for comparisons.^21^ An additional limitation could arise from the imputation of missing values. MICE is based on the assumption of missing at random (MAR). MAR implies that missing values do not depend on the unobserved but only on the observed data. As unobserved data are unknown, this assumption cannot be tested.^31, 42^ However, considering a high number of observed data can reduce the risk of bias.^31^

## CONCLUSION

The PARADISE intervention—a practice team-supported exposure training for panic disorder and agoraphobia in primary care—is likely to be cost effective, delivering high value for money.

## Electronic supplementary material

ESM 1 (DOCX 44.4 kb)

## Data Availability

The datasets analysed during the current study are available from the corresponding author on reasonable request.
