# Supplementary material for: Cost-effectiveness of Practice Team-Supported Exposure Training for Panic Disorder and Agoraphobia in Primary Care: a Cluster-Randomized Trial
Source: J Gen Intern Med. 2020 Jan 21;35(4):1120–6. doi: 10.1007/s11606-020-05658-9 (PMC7174430; doi:10.1007/s11606-020-05658-9)
Supplement: Supplementary file 1 — (DOCX 44.4 kb) [file 11606_2020_5658_MOESM1_ESM.docx]

**APPENDIX**

Table A1: Missing Values per time point and trial arm

| **Time Point** | **Variable** | **Description** | **Complete Sample** | **Control Group** | **Intervention Group** |
| --- | --- | --- | --- | --- | --- |
| T0 | Pat_Treat | Treatment group | 0% | 0% | 0% |
|  | Pat_Alter | Age at baseline | 0% | 0% | 0% |
|  | Pat_Sex | Sex at baseline | 0% | 0% | 0% |
|  | P03_rec_T0 | Partnership | 0% | 0% | 0% |
|  | P05_rec_T0 | Degree of education | 0% | 0% | 0% |
|  | AV1_T0 | Contacts to psychiatrists | 0.72% | 1.06% | 0.43% |
|  | AV2_T0 | Contacts to psychotherapists | 0.72% | 1.06% | 0.43% |
|  | AV3_T0 | Contacts to GPs | 2.86% | 1.06% | 4.35% |
|  | AV4_T0 | Contacts to other physicians | 0.95% | 1.59% | 0.43% |
|  | Besch0_T0 | Employed | 0% | 0% | 0% |
|  | Besch1_T0 | Sickness days | 5.25% | 5.29% | 5.22% |
|  | Besch2_T0 | Hours absent from work due to therapy | 10.98% | 11.11% | 10.87% |
|  | Besch3_T0 | Presenteeism | 4.30% | 2.12% | 6.09% |
|  | EQ1_0_T0 | EQ-5D: Mobility | 1.19% | 1.59% | 0.87% |
|  | EQ2_0_T0 | EQ-5D: Self-Care | 2.15% | 2.65% | 1.74% |
|  | EQ3_0_T0 | EQ-5D: Usual activities | 1.43% | 1.59% | 1.30% |
|  | EQ4_0_T0 | EQ-5D: Pain | 1.91% | 1.06% | 2.61% |
|  | EQ5_0_T0 | EQ-5D: Depression | 1.19% | 1.06% | 1.30% |
|  | EQ6_0_T0 | EQ VAS | 2.86% | 2.65% | 3.04% |
|  | HF111_T0 | Hours of professional help, received | 0.72% | 1.06% | 0.43% |
|  | HF121_T0 | Hours of informal care, received | 1.43% | 2.12% | 0.87% |
|  | Med_Kosten_Soma_T0 | Drug costs, somatic | 3.82% | 2.12% | 5.22% |
|  | Med_Kossten_Psych_T0 | Drug costs, psychopharmaceuticals | 3.82% | 2.12% | 5.22% |
|  | P04_T0 | Household size | 0.48% | 0.00% | 0.87% |
|  | P06_T0 | Years of education | 0.24% | 0.53% | 0.00% |
|  | Rent0_0_T0 | Retired | 0.95% | 1.06% | 0.87% |
|  | RESULT_BAI_T0 | Overall severity anxiety | 4.77% | 5.29% | 4.35% |
|  | RESULT_PHQ_T0 | Overall severity depression | 5.97% | 4.76% | 6.96% |
|  | RESULT_MIA_T0 | Overall severity of mobility inhibition | 0% | 0% | 0% |
|  | SB_OASIS1 | Frequency of anxiety | 1.67% | 1.59% | 1.74% |
|  | SB_OASIS2 | Intensity of anxiety | 1.67% | 1.59% | 1.74% |
|  | SB_OASIS3 | Frequency of avoidance behavior | 1.67% | 1.59% | 1.74% |
|  | SB_OASIS4 | Inhibition of anxiety | 1.91% | 2.12% | 1.74% |
|  | SB_OASIS5 | Loss of social contacts | 1.91% | 2.12% | 1.74% |
|  | SV1_T0 | Days in general hospital | 0.72% | 1.06% | 0.43% |
|  | SV2_T0 | Days in psychiatric hospital | 0.72% | 1.06% | 0.43% |
|  | SV3_T0 | Days in rehabilitation clinic | 0.72% | 1.06% | 0.43% |
| T1 | AV1_T1 | Contacts to psychiatrists | 18.85% | 13.23% | 23.48% |
|  | AV2_T1 | Contacts to psychotherapists | 18.85% | 13.23% | 23.48% |
|  | AV3_T1 | Contacts to GPs | 19.33% | 13.76% | 23.91% |
|  | AV4_T1 | Contacts to other physicians | 18.85% | 13.23% | 23.48% |
|  | Besch0_T1 | Employed | 16.95% | 11.64% | 21.30% |
|  | Besch1_T1 | Sickness days | 21.96% | 15.34% | 27.39% |
|  | Besch2_T1 | Hours absent from work due to therapy | 24.58% | 19.05% | 29.13% |
|  | Besch3_T1 | Presenteeism | 21.24% | 15.87% | 25.65% |
|  | EQ1_0_T1 | EQ-5D: Mobility | 20.05% | 14.81% | 24.35% |
|  | EQ2_0_T1 | EQ-5D: Self-Care | 19.81% | 14.81% | 23.91% |
|  | EQ3_0_T1 | EQ-5D: Usual activities | 19.09% | 13.76% | 23.48% |
|  | EQ4_0_T1 | EQ-5D: Pain | 20.05% | 14.29% | 24.78% |
|  | EQ5_0_T1 | EQ-5D: Depression | 19.57% | 13.76% | 24.35% |
|  | EQ6_0_T1 | EQ VAS | 19.81% | 14.29% | 24.35% |
|  | HF111_T1 | Hours of professional help, received | 19.09% | 13.23% | 23.91% |
|  | HF121_T1 | Hours of informal care, received | 19.09% | 13.23% | 23.91% |
|  | Med_Kosten_Soma_T1 | Drug costs, somatic | 22.20% | 15.87% | 27.39% |
|  | Med_Kosten_Psych_T1 | Drug costs, psychopharmaceuticals | 22.20% | 15.87% | 27.39% |
|  | Rent0_0_T1 | Retired | 18.85% | 13.23% | 23.48% |
|  | RESULT_BAI_T1 | Overall severity anxiety | 22.67% | 16.93% | 27.39% |
|  | RESULT_PHQ_T1 | Overall severity depression | 24.58% | 19.58% | 28.70% |
|  | RESULT_MIA_T1 | Overall severity of mobility inhibition | 0% | 0% | 0% |
|  | SV1_T1 | Days in general hospital | 19.09% | 13.23% | 23.91% |
|  | SV2_T1 | Days in psychiatric hospital | 19.09% | 13.23% | 23.91% |
|  | SV3_T1 | Days in rehabilitation clinic | 19.09% | 13.23% | 23.91% |
| T2 | AV1_T2 | Contacts to psychiatrists | 24.58% | 21.16% | 27.39% |
|  | AV2_T2 | Contacts to psychotherapists | 24.58% | 21.16% | 27.39% |
|  | AV3_T2 | Contacts to GPs | 30.07% | 26.98% | 32.61% |
|  | AV4_T2 | Contacts to other physicians | 24.58% | 21.16% | 27.39% |
|  | Besch0_T2 | Employed | 23.87% | 20.11% | 26.96% |
|  | Besch1_T2 | Sickness days | 26.25% | 22.22% | 29.57% |
|  | Besch2_T2 | Hours absent from work due to therapy | 27.21% | 23.81% | 30.00% |
|  | Besch3_T2 | Presenteeism | 25.78% | 20.63% | 30.00% |
|  | EQ1_0_T2 | EQ-5D: Mobility | 24.58% | 21.16% | 27.39% |
|  | EQ2_0_T2 | EQ-5D: Self-Care | 24.11% | 20.63% | 26.96% |
|  | EQ3_0_T2 | EQ-5D: Usual activities | 24.82% | 21.69% | 27.39% |
|  | EQ4_0_T2 | EQ-5D: Pain | 25.06% | 21.16% | 28.26% |
|  | EQ5_0_T2 | EQ-5D: Depression | 24.82% | 20.63% | 28.26% |
|  | EQ6_0_T2 | EQ VAS | 24.82% | 21.16% | 27.83% |
|  | HF111_T2 | Hours of professional help, received | 24.58% | 21.16% | 27.39% |
|  | HF121_T2 | Hours of informal care, received | 24.58% | 21.16% | 27.39% |
|  | Med_Kosten_Soma_T2 | Drug costs, somatic | 28.40% | 23.28% | 32.61% |
|  | Med_Kosten_Psych_T2 | Drug costs, psychopharmaceuticals | 28.40% | 23.28% | 32.61% |
|  | Rent0_0_T2 | Retired | 23.87% | 20.11% | 26.96% |
|  | RESULT_BAI_T2 | Overall severity anxiety | 26.01% | 23.28% | 28.26% |
|  | RESULT_PHQ_T2 | Overall severity depression | 27.92% | 25.40% | 30.00% |
|  | RESULT_MIA_T2 | Overall severity of mobility inhibition | 0% | 0% | 0% |
|  | SV1_T2 | Days in general hospital | 24.58% | 21.16% | 27.39% |
|  | SV2_T2 | Days in psychiatric hospital | 24.58% | 21.16% | 27.39% |
|  | SV3_T2 | Days in rehabilitation clinic | 24.58% | 21.16% | 27.39% |
| T1/IG | Pat_AT1 | First intervention session |  |  | 13.04% |
|  | Pat_AT2 | Second intervention session |  |  | 14.35% |
|  | Pat_AT3 | Third intervention session |  |  | 14.35% |
|  | Pat_AT4 | Fourth intervention session |  |  | 14.78% |
|  | Pat_ATzus | Additional intervention sessions |  |  | 15.65% |
|  | Pat_MFAtel | Number of telephone contacts (medical assistant) |  |  | 14.35% |
